# Prevalence of metabolic syndrome in Brazilian adults in the last 10 years: a systematic review and meta-analysis

**DOI:** 10.1186/s12889-022-12753-5

**Published:** 2022-02-16

**Authors:** Letícia Teixeira de Siqueira Valadares, Luiza Siqueira Barreto de Souza, Valdir Alves Salgado Júnior, Larissa de Freitas Bonomo, Leandro Roberto de Macedo, Maísa Silva

**Affiliations:** 1 Universidade Federal de Juiz de Fora, Campus Governador Valadares, Governador Valadares, Minas Gerais Brazil; 2 Department of Pharmacy, Universidade Federal de Juiz de Fora, Governador Valadares, Brazil; 3 Department of Economy, Universidade Federal de Juiz de Fora, Governador Valadares, Brazil; 4 Department of Basic Life Sciences, Universidade Federal de Juiz de Fora, Governador Valadares Campus, Avenida Moacir Paleta, nº 1167, no bairro São Pedro.CEP 35020-360, Governador Valadares, MG Brazil

**Keywords:** Prevalence, Metabolic syndrome, Meta-analysis, Brazil

## Abstract

**Background:**

A cluster of interconnected cardiometabolic risk factors characterizes metabolic Syndrome (MS). The prevalence of MS is increasing worldwide, but there is not a meta-analysis of this prevalence in the Brazilian population. We aimed to determine the prevalence of metabolic syndrome among adult general population in Brazil through a meta‑analysis study.

**Methods:**

Original research studies were searched at PubMed, Scopus, Web of Science, and SciELO databases, from 2011 to 2021. We used the Joanna Briggs Institute tool to assess the quality of included studies. The random effect model was used to estimate the pooled prevalence of MS. Subgroup and meta-regression analysis were conducted for explored heterogeneity and used the Funnel Plot and Egger’s test to assess publication bias. The study was performed based on the criteria of Preferred Reporting Items for Systematic Reviews and Meta-Analysis (PRISMA).

**Results:**

The search in electronic databases identified 1598 records. From this total, 26 studies were eligible to be included in the final analysis. The overall pooled prevalence among the general population of Brazil was 33% with high heterogeneity observed. By gender, the prevalences were 26% in males and 38% in females. By criteria that was used to define MS, the prevalence were 31% in NCEP ATP III, 25% in JIS, 37% in IDF/NHLBI/AHA/WHF/IAS/IASO and 33% in IDF criteria. The prevalence in different habitat was 34% in urban, 15% in rural, 28% in quilombola and 37% in indigenous. In different regions was 37% in the South, 30% in Southeast, 38% in North, 31% in Northeast and 39% in Midwest. The pooled prevalence of MS with age was < 45 years: 43% and ≥ 45 years: 42% and the prevalence based on year of study implementation was 31% in 2015–2019, 35% in 2010–2014 and 28% in 2005–2009. There were no statistically significant differences between subgroups. Most of the studies showed high quality assessment criteria’s except adequate sample size criteria and many studies participants were not sampled in an appropriate way.

**Conclusions:**

Our review indicates a high prevalence of MS in the healthy Brazilian adult population, when compared to others countries and with a world estimate.

**Supplementary Information:**

The online version contains supplementary material available at 10.1186/s12889-022-12753-5.

## Background

Metabolic syndrome (MS) is a complex disorder characterized by the association of cardiovascular risk factors and insulin resistance [1]. The components that define MS include hyperglycemia, hypertension, high triglyceride levels, low high density lipoprotein (HDL) cholesterol levels and abdominal obesity [2].

Most of these components are used as diagnostic criteria by some guidelines, such as the International Diabetes Federation (IDF) [3] and the National Cholesterol Education Program (Adult Treatment Panel III) (NCEP-ATPIII) [4], in addition to the World Health Organization (WHO) [5]. Generally, studies that used more than one guideline to define the prevalence of MS, observed a discrepancy in the results found [6, 7]. This difference occurs because there are divergent points between the assessment factors used by each of the definitions [8]. In the case of the WHO and the NCEP-ATPIII, for example, the main difference is that the former considers microalbuminuria and obesity to be diagnostic factors for the metabolic syndrome, and the NCEP-ATPIII requires that, among the components used for diagnosis, for a confirmation of a case of MS, at least three are altered [9]. Unlike the NCEP-ATPIII and IDF criteria, the WHO also considers the presence of type 2 diabetes mellitus (DM2) a mandatory factor for diagnosis which, probably, when compared with the other two methods, makes this one find a smaller number of MS patients [10].

Regardless of the criteria used for diagnosis, it is well accepted that the prevalence of MS is increasing at epidemic proportions in developed and developing countries [11]. The global prevalence of this condition in the adult population is estimated at around 20 to 25% [12]. In relation to Latin America, the general prevalence found was similar, around 24.9%, with a greater predominance of women and in the age group above 50 years old [13]. In Brazil, the prevalence was estimated in 2013, in the adult population at around 28.9 and 29.6% [14].

MS demands high expenses of the health system, in addition to causing considerable damage to the quality of life of patients, and is therefore considered a serious public health problem worldwide [15, 16]. In this regard, studies demonstrate a high prevalence of MS in the general population and particularly among participants with pre-diabetes, DM2 and patients at high risk for CVD. Therefore, screening for MS in health centers can identify patients at higher risk for these conditions, and multifactorial interventions can benefit this population [6]. Thus, it emphasizes the importance of studies on the prevalence of the syndrome to assist in designing and directing measures to prevent the development of this condition. Several systematic reviews and meta-analyses on the prevalence of MS have been published in various parts of the world [17–19], including a systematic review in Brazilian adults in 2013 [14]. However, as far as we know, no quantitative data analysis (meta-analysis) of this prevalence in Brazil was evaluated. Therefore, our objective was to develop a systematic review and meta-analysis summarizing available epidemiological data on the prevalence of MS among adults in the Brazilian population.

## Methods

### Data sources and searches

The present systematic review and meta-analysis was performed in accordance with the Preferred Reporting Items for Systematic Review and Meta-analysis (PRISMA) guidelines [20] and the PRISMA 2020 check list [21]. The review has been registered at PROSPERO (www.crd.york.ac.uk/prospero/), registration number CRD42021241890. A literature search was carried out to identify prevalence of MS in Brasilian adults. The studies were identified through systematically search at PubMed, Scopus, Web of Science, and SciELO, for relevant studies published before april 2021. “The following keywords were used in combination: ‘‘metabolic syndrome” or Syndrome X or MS, and ‘‘prevalence,’’ and ‘‘Brazil.’’ No language restrictions were imposed. A manual review of the reference lists, in gray literature and research in unpublished data was also conducted.

### Study selection

The search was performed independently by three authors (LTSV, LSBS, and VASJ). This reviewers independently identified potentially eligible articles by performing an initial screen of titles and abstracts. All potentially relevant titles and abstracts were selected for full text examination. Any discrepancies among the reviewers were resolved through consensus. Then, the following inclusion criteria were applied: (I) original type studies (II) studies that were conducted among 18 years of age or older [22] and reportedly healthy individuals of both sexes (III) There were no restrictions geographic region (urban, rural) and (IV) to define MS, studies that used any defined criteria to determine the prevalence of MS.

The exclusion criteria for our study were as follows: (I) the reviews and letters to the editors, (II) studies that used animal models or in vitro, (III) studies performed outside of Brazil, (IV) the study population comprising individuals who were reported to have other health complications, (V) studies with incomplete information [6] or in a specific population.

### Data extraction

The three investigators extracted the data independently. The following information collected from each study was: first author’s name, year of publication, gender, age range, city and region of study and area in which the study was carried out, population, study design, criteria for diagnosis of metabolic syndrome, and the prevalence of metabolic of syndrome and its components.

### Quality of studies

Study quality was assessed independently and blindly by three reviewers using the Joanna Briggs Institute tool for cross-sectional studies (JBI Critical Appraisal Checklist for Analytical Cross Sectional Studies) [23]. This tool consists of a checklist of nine items, which determine the adequacy of the inclusion criteria; sample description; were study participants recruited in an appropriate way, was the sample size adequate, were the study subjects and setting described in detail, sufficient coverage of the identified sample, standardization of diagnostic criteria, reliability and validity of the results, use of adequate statistical analysis, and response rate adequate. The answer options were yes, no, unclear and not applicable. The divergences in the analysis were resolved by consensus.

### Statistical analysis

The meta-analysis was performed using R software (R Foundation for Statistical Computing, Vienna, Austria, URL http://www.R-project.org, 2020). The prevalence of MS reported in the selected studies among healthy Brasilian adult populations was analyzed based on different diagnostic criteria used. In each study, we extracted the total number of participants and the number of individuals with the outcome. If one of these data was not provided by the article, we obtained this value through the prevalence of metabolic syndrome.

We used random effect models to calculate pooled prevalence and 95% confidence intervals. Inter-study heterogeneity was explored quantitatively using Cochran's Q and ${I}^{2}$ tests [24]. In this regard, an ${I}^{2}$ of 50% and 75% indicated substantial and considerable heterogeneity, respectively. We used the fixed effect for ${I}^{2}$< 50% (low heterogeneity). We explored sources of heterogeneity by comparing MS prevalence across subgroups defined by several study-level characteristics and meta-regression analyses according to the year of implementation of study and age of the participants. We assessed the presence of publication bias graphically using the funnel plot. Publication bias also was evaluated using Egger’s Test, the significance level was set at a P value of less than 0.05 [25].

## Results

**Fig. 1:**
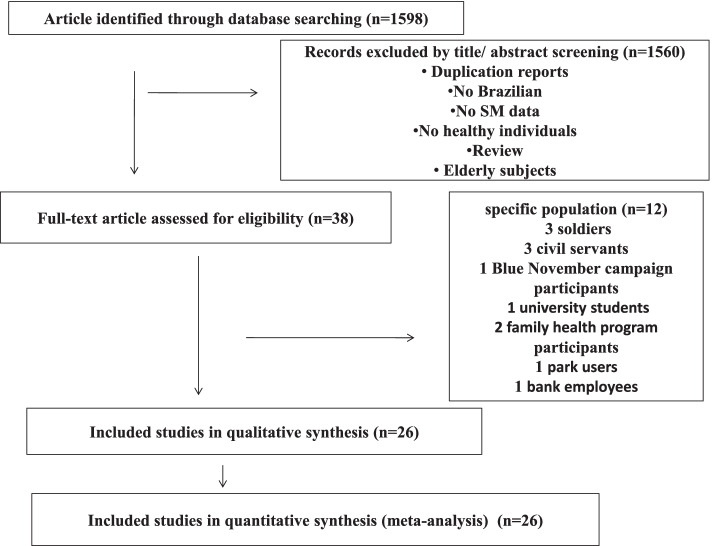
Flow diagram of studies included in the systematic review

The flow of the literature search is shown in Fig. 1. An initial search of the electronic databases identified 1598 records. Overall, 1560 records were excluded that did not meet the inclusion criteria. Therefore, 38 studies were assessed for eligibility through full-text reading. Of these, 12 studies were excluded for consisting of specific population. Finally, 26 studies were selected for systematic review and meta-analysis.

### Characteristics of the included studies

The characteristics of studies published between 2011 and 2021 on the prevalence of MS in Brazil are included in Table 1. Most of the studies were performaded in urban populations [6, 7, 26–39]. All included studies were cross-sectional design. One study carried out only on female participants [30]. Eight studies used the criteria for diagnosing metabolic syndrome proposed by the NCEP-ATP III [30, 35, 36, 40–44]; three the criteria of the IDF [31, 38, 39]; ten studies used International Diabetes Federation Task Force on Epidemiology and Prevention; National Heart, Lung, and Blood Institute; American Heart Association; World Heart Federation; International Atherosclerosis Society; and International Association for the Study of Obesit (IDF/NHLBI/AHA/WHF/IAS/ IASO) [26–28, 32–34, 37, 45–47]; two studies used Joint Interim Statement (JIS) [29, 48]; one study used NCEP ATP III and IDF criteria [7]; one study used modified NCEP, IDF and JIS criteria for diagnosing MS [6]; and one study did not present the criteria it used for the diagnosis of MS [49].

**Table 1:** Characteristics of studies that evaluated the prevalence of metabolic syndrome in the Brasilian population

Study and Year Published	Age Range	Sample Size (male/female)	City and region	Population	Study Design	Criteria for Diagnosis of SM	Overall Prevalence of SM (%)	Prevalence of individual components of SM (%)
Gouveia et al. 2021 [26]	59.8 ± 19.7	910(341/569)	Fonte Boa, Apuí, and Manaus – Amazonas state	Adults and Older Adults – Urban	cross-sectional study	IDF/NHLBI/AHA/WHF/IAS/IASO	47.5 (39.6 men, 52.2 women)	Elevated waist circumference: 56.1; High blood pressures: 53.8; Elevated fasting blood glucose: 30.9; Low HDL cholesterol: 39.8; High triglyceride: 37.2
Oliveira et al. 2020 [27]	45.6	8199	Pesquisa Nacional de Saúde de 2013	Urban	analytical cross-sectional study	IDF/NHLBI/AHA/WHF/IAS/IASO	38.4 (34.6 men, 41.8 women)	Elevated waist circumference: 65.5; High blood pressures: 32.3; Elevated fasting blood glucose: N/A; Low HDL cholesterol: 49.4; High triglyceride: N/A
Santos et al. 2020 [28]	25—65 years	818 (349/469)	Florianópolis, Santa Catarina state	Urban	cross-sectional population-based study	IDF/NHLBI/AHA/WHF/IAS/IASO	30.9 (36.1 men; 27.2 women)	Elevated waist circumference: 50.1; High blood pressures: 66.5; Elevated fasting blood glucose: 16.8; Low HDL cholesterol: 37.4; High triglyceride: 20.2
do Vale Moreira et al. 2020 [6]	≥ 20 years	714 (242/472)	Pindoretama, Ceará state	Urban	cross-sectional population-based study	Modified NCEP, IDF and JIS	JIS = 36.1 IDF = 35.1 NCEP = 29.5	N/A
Moreira et al. 2020 [30]	50.1 ± 5.5	419 (women)	Parnamirim—Rio Grande do Norte state	middle-aged women – Urban	cross-sectional study	NCEP ATP III	65.6	Elevated waist circumference: 73.5; High blood pressures: 60.9; Elevated fasting blood glucose: 16.9; Low HDL cholesterol: 63.0; High triglyceride: 40.8
Carvalho et al. 2019 [29]	23.9 years	2017(946/1071)	Ribeirão Preto, São Paulo state	Urban	cross-sectional study	JIS	12.2 (18.9 men; 6.3 women)	N/A
Luisi et al. 2019 [49]	≥ 18 years	193(74/119)	Tocantins state	Quilombola communities	observational cross-sectional study	N/A	32.12 (17,6 men; 41,2 women)	Elevated waist circumference: 58.0; High blood pressures: 41.5; Elevated fasting blood glucose: 35.2; Low HDL cholesterol: 52.3; High triglyceride: 15.5
Mulatinho et al. 2019 [31]	24—59 years	375(118/257)	Fernando de Noronha Archipelago, Pernambuco state	Urban	Cross-sectional study	IDF	11.97 (3.72 men, 8.24 women)	Elevated waist circumference: 70.4; High blood pressures: 0; Elevated fasting blood glucose: 19.15; Low HDL cholesterol: 21.01; High triglyceride: 19.68
Mussi et al. 2019 [48]	45 years	842(325/517)	Guanambi, Bahia state	Quilombola communities	cross-sectional population-based study	JIS	25.8 (20.9 men, 28.8 women)	N/A
Ramires et al. 2018 [32]	≥ 18 years	59,402 (25.920/ 33.482)	Brazilian Adult Population: National Health Survey – 2013	Urban	household-based cross-sectional	IDF/NHLBI/AHA/WHF/IAS/IASO	8,9 (7.5 men, 10.3 women)	Elevated waist circumference: 65.2; High blood pressures: 40.7; Elevated fasting blood glucose: 7.1; Low HDL cholesterol: N/A; High triglyceride: N/A
França et al. 2016 [33]	42.2 ± 16.3	787 (188/599)	Marajó Archipelago, Para state	Urban	cross-sectional population-based	IDF/NHLBI/AHA/WHF/IAS/IASO	34.1 (29.8 men, 35.4 women)	Elevated waist circumference: 55.3; High blood pressures: 47.6; Elevated fasting blood glucose: 24.3; Low HDL cholesterol: 56.2; High triglyceride: 19.9
Bortoletto et al. 2016 [34]	54.5 ± 10.3	959 (426/533)	Cambé, Paraná state	≥ 40 years adults—Urban	cross-sectional population-based	IDF/NHLBI/AHA/WHF/IAS/IASO	53.7 (48.4 men, 58 women)	N/A
Soares et al. 2015 [45]	42.7 ± 19.1	932 (457/475)	Indian reservations, Mato Grosso state	Xavante indigenous	cross‑sectional study	IDF/NHLBI/AHA/WHF/IAS/IASO	66.1 (55.6 men, 76.2 women)	Elevated waist circumference: 92.6; High blood pressures: 41.4; Elevated fasting blood glucose: 76.4; Low HDL cholesterol: 86.6; High triglyceride: 71.15
Martini et al. 2014 [35]	≥ 20 years	1112(468/644)	Ourinhos, São Paulo state	Urban	observational cross-sectional study	NCEP ATP III	24.1 (27.8 men, 20.3 women)	Elevated waist circumference: 36.7; High blood pressures: 46.2; Elevated fasting blood glucose: 13.9; Low HDL cholesterol: 45.4; High triglyceride: 23.1
Moreira et al. 2014 [36]	55.0 ± 14.7	1369(667/702)	Population in Brazil	Urban	cross-sectional, population based study	NCEP ATP III	22.7 ( 23.3 men, 22.7 women)	N/A
Pimenta et al. 2013 [40]	≥ 18 years	491 (246/245)	Virgem das Graças and Caju, in the rural areas of the municipalities of Ponto dos Volantes and Jequitinhonha, respectively, Minas Gerais state	Rural	cross-sectional population-based	NCEP ATP III	14.9 (6.5 men, 23.3 women)	Elevated waist circumference: 11.6; High blood pressures: 59.7; Elevated fasting blood glucose: 10.6; Low HDL cholesterol: 44.1; High triglyceride: 15.2
da Rocha et al. 2013 [41]	55.5 ± 13.23	73 (23/50)	Village Pinhalzinho located at Planalto/Nonoai City, Rio Grande do Sul state	Kaingang indigenous	cross-sectional descriptive and analytical study	NCEP ATP III	23.3 (47.1 men, 52.9 women)	N/A
Dutra et al. 2012 [37]	≥ 18 years	2130 (586/1544)	Brasilia, Federal District	Urban	cross-sectional, population based study	IDF/NHLBI/AHA/WHF/IAS/IASO	32 (30.9 men, 33 women)	N/A
Santos et al. 2012 [46]	38 ± 14.8	162 (98/64)	Medial region of the Xingu Indigenous Park, Mato Grosso state	Khisêdjê indigenous	cross-sectional study	IDF/NHLBI/AHA/WHF/IAS/IASO	27.8 (19.4 men, 40.6 women)	Elevated waist circumference:37.4; High blood pressures: 6.8; Elevated fasting blood glucose: 12.2; Low HDL cholesterol: 66.2; High triglyceride: 43.5
Gomes et al. 2012 [38]	57 ± 16	131 (54/77)	Community of Mombuca/Guatapara, São Paulo state	Japanese- Brazilian—Urban	cross-sectional study	IDF	35.8 (36.2 men, 63.8 women)	Elevated waist circumference: N/A; High blood pressures: 46.6; Elevated fasting blood glucose: N/A; Low HDL cholesterol: 44.3; High triglyceride: 26.7
Gronner et al. 2011 [7]	30 – 79 years	1116 (396/720)	São Carlos, São Paulo state	Urban	cross-sectional population-based study	NCEP-ATP III and IDF	ATP III 40.5 (36.1 men; 42.9 women) IDF 48.1 (49.2 men; 47.5 women)	Elevated waist circumference: 56.2 (NCEP criteria) and 72.6 (IDF criteria); High blood pressures: 59.2; Elevated fasting blood glucose: 13.3; Low HDL cholesterol: 76.3; High triglyceride: 16.8
da Rocha et al. 2011 [42]	≥ 40 years	150(67/83)	Porto Alegre e Planalto/Nonoai, Rio Grande do Sul state	Kaingang e Guarani indigenous	cross-sectional, descriptive and analytical	NCEP-ATPIII	65.3 (40.3 men / 85 women)	Elevated waist circumference: 87.6; High blood pressures: 82.5; Elevated fasting blood glucose: 86; Low HDL cholesterol: 72,3; High triglyceride: 85.5
Oliveira et al. 2011 [47]	36 ± 1	606 (268/338)	Jaguapiru village, Dourados, Mato Grosso do Sul state	Indigenous population	cross-sectional study	IDF/NHLBI/AHA/WHF/IAS/IASO	35.7 (26.1 men / 43.4 women)	Elevated waist Circumference: 60.9; High blood pressures: 40.3; Elevated fasting blood glucose: 11.4; Low HDL cholesterol: N/A; High triglyceride: N/A
Anjos et al. 2011 [43]	32 years	82(33/49)	Cândido de Abreu, state Paraná	Kaingang Indigenous	cross-sectional study	NCEP-ATPIII	11(0 men, 18.4 women)	Elevated waist Circumference: 37.8; High blood pressures: 26.8; Elevated fasting blood glucose: 9.4; Low HDL cholesterol: 13.4; High triglyceride: 11
Silva et al. 2011 A [39]	20 – 64 years	287 (73/214)	Metropolitan region of Sao Paulo, São Paulo state	Urban	descriptive and analytical study cross-section	IDF	36.6	N/A
Silva et al. 2011 B [44]	N/A	246 (91/155)	Inhaumas, district of Santa Maria da Vitória, Bahia state	Rural	cross-sectional study	NCEP-ATPIII	15.4 (11.9 men, 17.5 women)	N/A

*IDF/NHLBI/AHA/WHF/IAS/ IASO* International Diabetes Federation Task Force on Epidemiology and Prevention; National Heart, Lung, and Blood Institute; American Heart Association; World Heart Federation; International Atherosclerosis Society; and International Association for the Study of Obesit, *IDF* International Diabetes Federation, *JIS* Joint Interim Statement, *NCEP-ATP III* National Cholesterol Education Program Adult Treatment Panel III, *N/A* information not available

The studies selected in this systematic review comprised 84,522 subjects, 57.5% of whom were women and 42.5% men. The prevalence of metabolic syndrome is reported by all the studies ranged from 8.9% to 66.1%. Most of the studies where participants were both male and female, reported prevalence data not only for all but also for males and females separately. Many studies presented prevalence of individual components of MS [7, 26–28, 30–33, 35, 38, 40, 42, 43, 45–47, 49]; The component with the highest prevalence was increased waist circunference (WC), ranging from 37.8% to 92.6% [26, 27, 30–32, 42, 43, 45, 47, 49] followed by high blood pressures, ranging from 46.2% to 66.5% [28, 35, 40].

### Analysis of quality of studies

The quality of the studies was assessed according to the set of criteria based on JBI guidance and are summarized in Table 2. A set of nine criteria was used to assess the quality of the studies. The sample frame was appropriate to address a target population in almost all articles with one exception [35]. Fourteen study participants were sampled appropriately [6, 7, 27, 28, 31–34, 36, 37, 39, 43, 47, 48]. The sample size was adequate in 19 studies [6, 7, 26, 27, 29–32, 34–36, 40, 41, 44–49]. Study subjects and setting was described in detail in all articles. The data analysis was conducted with sufficient coverage of the identified sample in 77% studies [6, 7, 26, 27, 29, 30, 32, 34–36, 38, 40, 42–49]. Valid methods were used of identify of the condition in almost all articles with one exception [49]. The condition was measured in a standard and reliable way for all participants and there was an appropriate statistical analysis in all the studies. The response rate was adequate and, if not, the low response rate was adequately managed in almost all articles with two exceptions [34, 47].

**Table 2:** Study quality assessment of studies that evaluated the prevalence of metabolic syndrome in the brazilian population

**Study**	1- Was the sample frame appropriate to address the target population?	2- Were study participants sampled in an appropriate way?	3- Was the sample size adequate?	4- Were the study subjects and the setting described in detail?	5- Was the data analysis conducted with sufficient coverage of the identified sample?	6- Were valid methods used for the identification of the condition?	7- Was the condition measured in a standard, reliable way for all participants?	8- Was there appropriate statistical analysis?	9- Was the response rate adequate, and if not, was the low response rate managed appropriately?
Gouveia et al. 2021 [26]	Yes	No	Yes	Yes	Yes	Yes	Yes	Yes	Yes
Oliveira et al. 2020 [27]	Yes	Yes	Yes	Yes	Unclear	Yes	Yes	Yes	Yes
Santos et al. 2020 [28]	Yes	Yes	No	Yes	Yes	Yes	Yes	Yes	Yes
do Vale Moreira et al. 2020 [6]	Yes	Yes	Yes	Yes	Yes	Yes	Yes	Yes	Yes
Moreira et al. 2020 [30]	Yes	No	Yes	Yes	Yes	Yes	Yes	Yes	Yes
Carvalho et al. 2019 [27]	Yes	Unclear	Yes	Yes	Yes	Yes	Yes	Yes	Yes
Luisi et al. 2019 [49]	Yes	No	Yes	Yes	Yes	No	Yes	Yes	Yes
Mulatinho et al. 2019 [31]	Yes	Yes	Yes	Yes	No	Yes	Yes	Yes	Yes
Mussi et al. 2019 [48]	Yes	Yes	Yes	Yes	Yes	Yes	Yes	Yes	Yes
Ramires et al. 2018 [32]	Yes	Yes	Yes	Yes	Yes	Yes	Yes	Yes	Yes
França et al. 2016 [33]	Yes	Yes	No	Yes	No	Yes	Yes	Yes	Yes
Bortoletto et al. 2016 [34]	Yes	Yes	Yes	Yes	Yes	Yes	Yes	Yes	No
Soares et al. 2015 [45]	Yes	Unclear	Yes	Yes	Yes	Yes	Yes	Yes	Yes
Martini et al. 2014 [35]	No	No	Yes	Yes	Yes	Yes	Yes	Yes	Yes
Moreira et al. 2014 [36]	Yes	Yes	Yes	Yes	Yes	Yes	Yes	Yes	Yes
Pimenta et al. 2013 [40]	Yes	Unclear	Yes	Yes	Yes	Yes	Yes	Yes	Yes
da Rocha et al. 2013 [41]	Yes	No	Yes	Yes	No	Yes	Yes	Yes	Yes
Dutra et al. 2012 [37]	Yes	Yes	No	Yes	No	Yes	Yes	Yes	Yes
Santos et al. 2012 [46]	Yes	Unclear	Yes	Yes	Yes	Yes	Yes	Yes	Yes
Gomes et al. 2012 [38]	Yes	Unclear	No	Yes	Yes	Yes	Yes	Yes	Yes
Gronner et al. 2011 [7]	Yes	Yes	Yes	Yes	Yes	Yes	Yes	Yes	Yes
da Rocha et al. 2011 [42]	Yes	Unclear	Unclear	Yes	Yes	Yes	Yes	Yes	Yes
Oliveira et al. 2011 [47]	Yes	Yes	Yes	Yes	Yes	Yes	Yes	Yes	No
Anjos et al. 2011 [43]	Yes	Yes	No	Yes	Yes	Yes	Yes	Yes	Yes
Silva et al. 2011 A [39]	Yes	Yes	No	Yes	No	Yes	Yes	Yes	Yes
Silva et al. 2011 B (44)	Yes	Unclear	Yes	Yes	Yes	Yes	Yes	Yes	Yes

### General prevalence of MS and analysis based on the gender of study participants

**Fig. 2:**
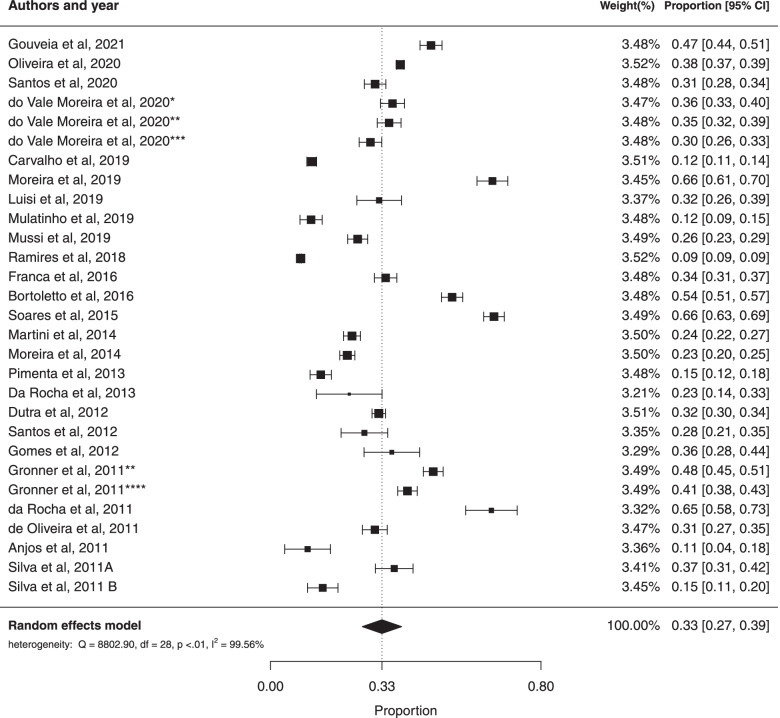
Forest plot of prevalence of metabolic syndrome in Brazilian population. * prevalence according to the JIS criteria, ** prevalence according to the IDF criteria, *** prevalence according to the modified NCEP_ATPIII criteria and **** prevalence according to the NCEP-ATPIII criteria

**Fig. 3:**
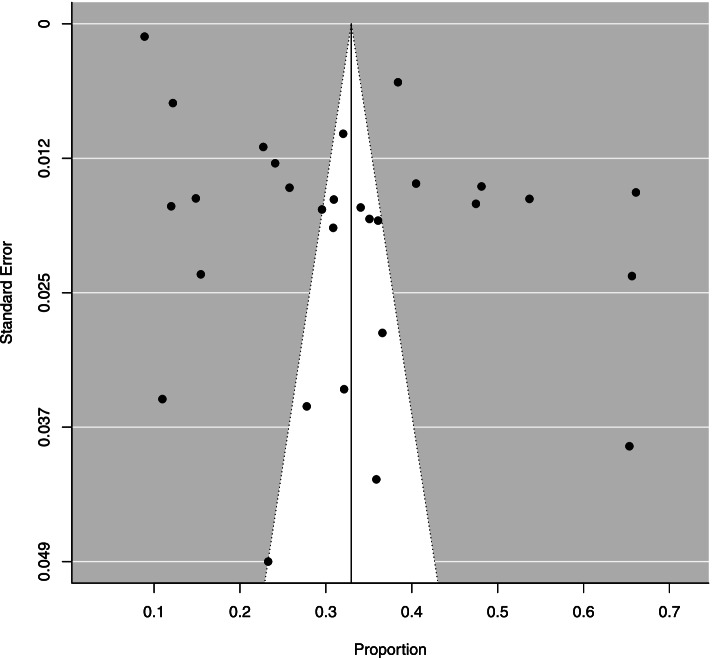
Funnel plot of the studies that evaluated the prevalence of metabolic syndrome in Brazilian population

To calculate the general prevalence, a meta-analysis was performed with the 26 studies that reported the prevalence of MS in Brazilian adults, using the random effects model. The general prevalence estimate was 33% (95% CI: 27%; 39%). There was a large amount of heterogeneity in the prevalence of metabolic syndrome (${I}^{2}$= 99.56%; Cochran Q-statistic *p* < 0.01; Fig. 2). In the funnel graph, there is an asymmetry, which suggests a possible publication bias (Fig. 3), however Egger’s test (*p* = 0.4851) suggested no significant publication bias.

**Fig. 4:**
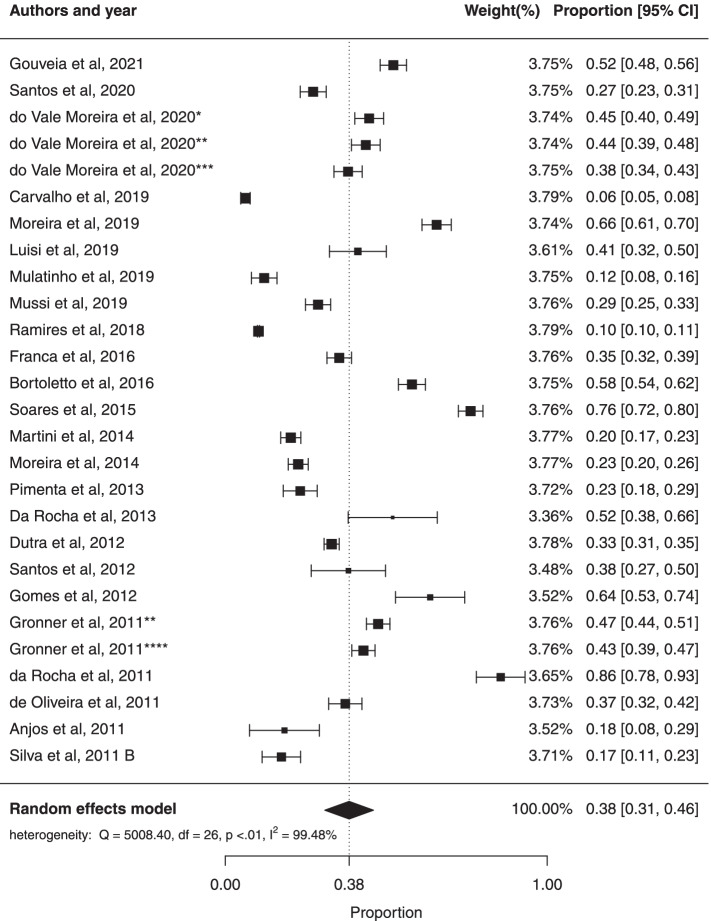
Forest plot of prevalence of metabolic syndrome in adult males in Brazilian population

**Fig. 5:**
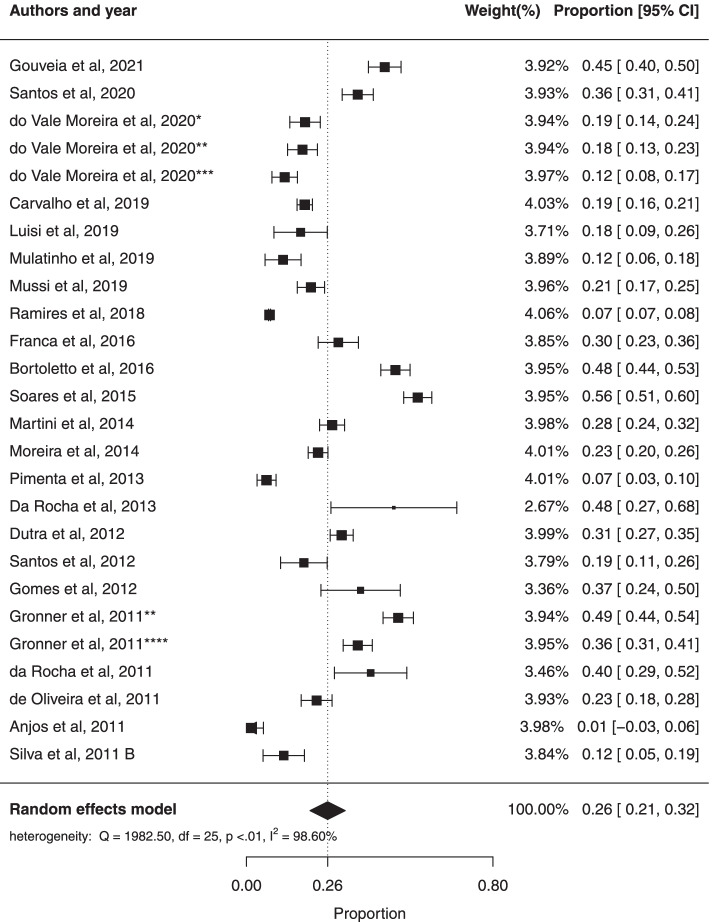
Forest plot of prevalence of metabolic syndrome in adult females in Brazilian population

The prevalence of MS in female and male was respectively 38% (95% CI: 31%; 46%) and 26% (95% CI: 21%; 32%). However, there was no statistical difference between the two groups. There was significant heterogeneity (${I}^{2}$= 99.48%; Cochran Q-statistic *p *< 0.01; Fig. 4) in the prevalence of MS in females and in male (${I}^{2}$= 98.60%; Cochran Q-statistic *p* < 0.01; Fig. 5). The results of this study suggested no significant publication bias using Egger test for female (*p* = 0.0992) and male (*p* = 0.0589).

### Subgroup analysis

#### Subgroup analysis based on criteria used to define metabolic syndrome

**Fig. 6:**
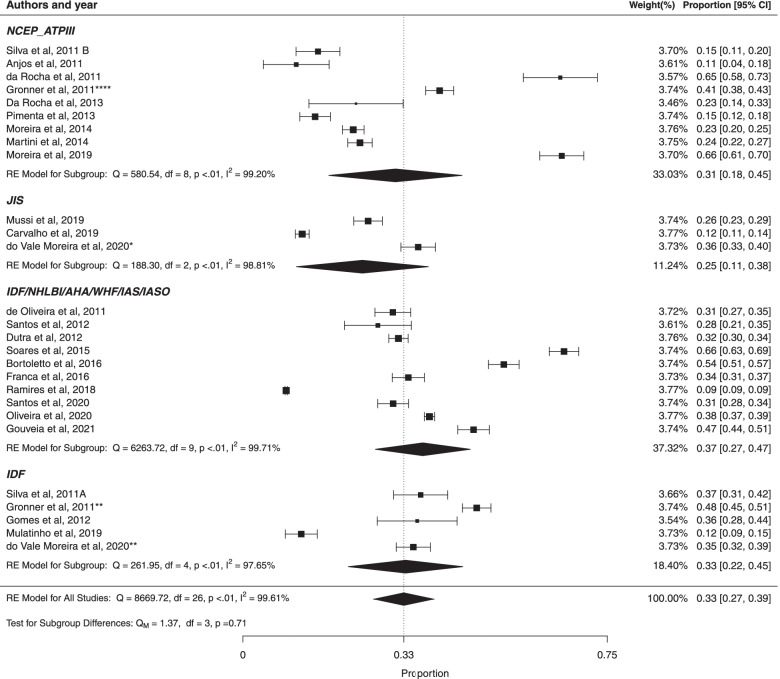
Forest plot of prevalence according criteria used to define metabolic syndrome in Brazilian population. * prevalence according to the JIS criteria, ** prevalence according to the IDF criteria and **** prevalence according to the NCEP-ATPIII criteria

Studies that used the NCEP-ATP III criteria to define MS had the pooled prevalence of metabolic syndrome of 31% (95% CI: 18%; 45%) with high heterogeneity (${I}^{2}$ = 99.20%; Cochran Q-statistic *p* < 0.01; Fig. 6). The pooled prevalence of metabolic syndrome of studies that used JIS criteria to diagnose metabolic syndrome was 25% (95% CI: 11%; 38%) with high heterogeneity (${I}^{2}$ = 98.81%; Cochran Q-statistic *p* < 0.01). The weighted pooled prevalence of metabolic syndrome of studies that used IDF/NHLBI/AHA/WHF/IAS/IASO criteria was 37% (95% CI: 27%; 47%), with with high heterogeneity (${I}^{2}$ = 99.71%; Cochran Q-statistic *p* < 0.01). The prevalence of MS in studies that used the IDF criteria was 33% (95% CI: 22%; 45%), with high heterogeneity (${I}^{2}$ = 97.65%; Cochran Q-statistic *p* < 0.01) There was not statistically significant difference between studies based on diagnostic criteria (*p* = 0.71). In addition, there was high heterogeneity in prevalence estimates across studies (all heterogeneity *p* < 0.01). The results of this study suggested no significant publication bias using Egger test for IDF/NHLBI/AHA/WHF/IAS/IASO criteria (*p* = 0.5906), NCEP – ATPIII (*p* = 0.7054) and IDF (*p* = 0.8432), however, the results of this study indicated the possibility of statistically significant bias using Egger test for JIS criteria (*p* < 0.001).

#### ***Subgroup ***analysis*** based on habitat of study participants***

**Fig. 7:**
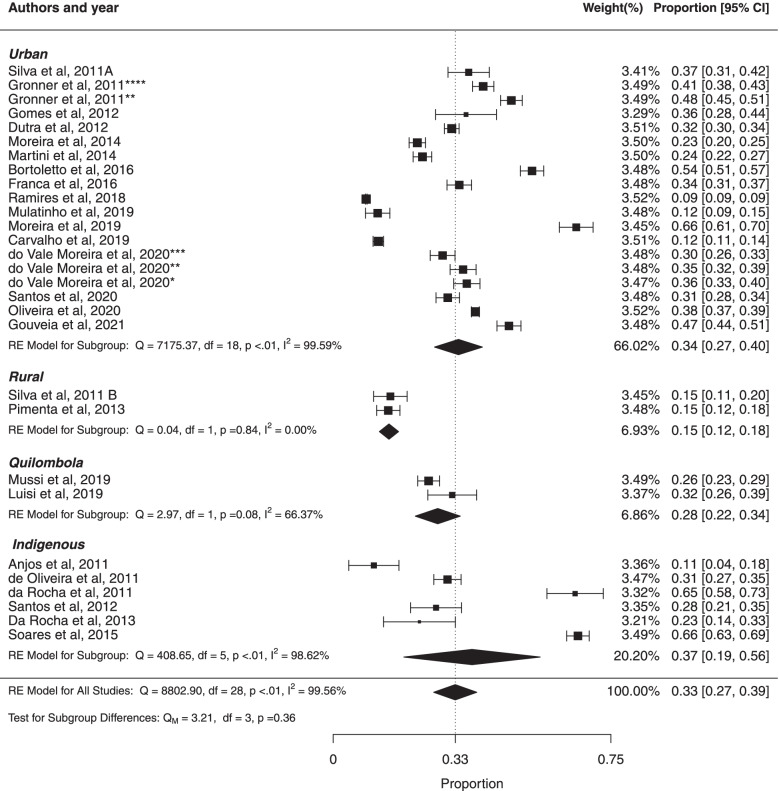
Forest plot of prevalence of metabolic syndrome according habitat of study participants in Brazilian population. * prevalence according to the JIS criteria, ** prevalence according to the IDF criteria, *** prevalence according to the modified NCEP_ATPIII criteria and **** prevalence according to the NCEP-ATPIII criteria

The pooled prevalence of MS in the population living in urban, rural, quilombola and indigenous areas were respectively (34%, 95% CI: 27%; 40%), (15%, 95% CI: 12%; 18%), (28%, 95% CI: 22%; 34%), and (37%, 95% CI: 19%; 56%). There was high heterogeneity in studies: in urban area (${I}^{2}$ = 99.59%; Cochran Q-statistic *p* < 0.01; Fig. 7), in quilombola area (${I}^{2}$ = 66.37%; Cochran Q-statistic *p* < 0.01) and in indigenous area (${I}^{2}$ = 98.62%; Cochran Q-statistic *p* < 0.01). There was not statistically significant difference between studies based on habitat (*p* = 0.36). In addition, there was high heterogeneity in prevalence estimates across studies (*p* < 0.01). The results of this study suggested no significant publication bias using Egger test for urban area (*p* = 0.0684) and indigenous area (*p* = 0.4279). Egger's test was not performed in the quilombola and rural subgroups because the number of studies included in these subgroups was small.

#### ***Subgroup analysis ***based*** on Brazilian regions of study participants***

**Fig. 8:**
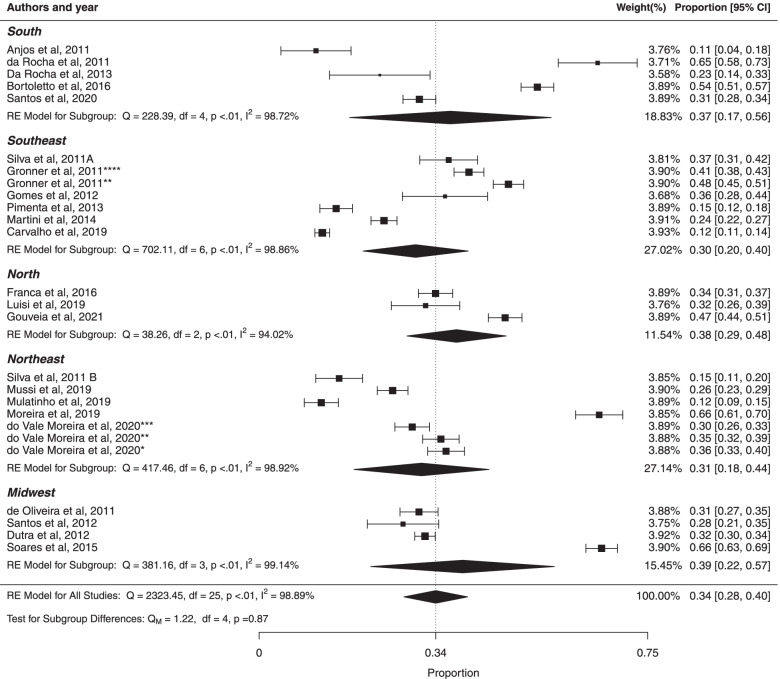
Forest plot of prevalence of metabolic syndrome according regions of study participants in Brazilian population. * prevalence according to the JIS criteria, ** prevalence according to the IDF criteria, *** prevalence according to the modified NCEP_ATPIII criteria and **** prevalence according to the NCEP_ATPIII criteria

The pooled prevalence of MS in the Brazilian population in the South, Southeast, North, Northeast and Midwest regions were respectively (37%, 95% CI: 17%; 56%), (30%, 95% CI: 20%; 30%), (38%, 95% CI: 29%; 48%), (31%, 95% CI: 18%; 44%) and (39%, 95% CI: 22%; 57%). There was high heterogeneity in South region (${I}^{2}$ = 98.72%; Cochran Q-statistic *p* < 0.01; Fig. 8), in Southeast region, (${I}^{2}$ = 98.86%; Cochran Q-statistic *p* < 0.01), in North region (${I}^{2}$ = 94.02%; Cochran Q-statistic *p* < 0.01); in Northeast region (${I}^{2}$ = 98.92%; Cochran Q-statistic *p* < 0.01) and in Midwest regions (${I}^{2}$ = 99.14%; Cochran Q-statistic *p* < 0.01). There was not statistically significant difference between studies based on regions (*p* = 0.87). In addition, there was high heterogeneity in prevalence estimates across studies (*p* < 0.01). The results, using Egger test, suggested no significant publication bias for South (*p* = 0.7032), Southeast (*p* = 0.3542), North (*p* = 0.4542), Northeast (*p* = 0.3004) and Midwest regions (*p* = 0.5850).

#### ***Subgroup ***analysis*** based on age of study participants***

**Fig. 9:**
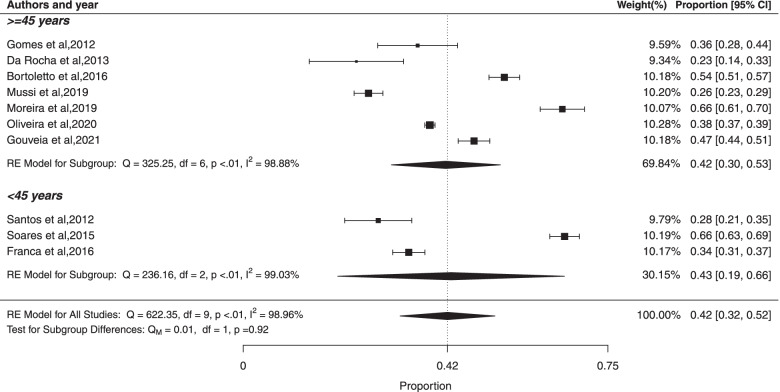
Forest plot of prevalence of metabolic syndrome according age of study participants in Brazilian population. * prevalence according to the JIS criteria, ** prevalence according to the IDF criteria, *** prevalence according to the modified NCEP_ATPIII criteria and **** prevalence according to the NCEP_ATPIII criteria

The pooled prevalence of MS among studies with participants 45 years of age or older was 42% (95% CI: 30%; 53%) with high heterogeneity (${I}^{2}$ = 98.88%; Cochran Q-statistic *p* < 0.01; Fig. 9). The studies that the participants had less than 45 years old, the pooled prevalence of MS was 43% (95% CI: 19%; 66%), with high heterogeneity (${I}^{2}$ = 99.03%; Cochran Q-statistic *p* < 0.01). There was not statistically significant difference between studies based on age of participants (*p* = 0.92). In addition, there was high heterogeneity in prevalence estimates across studies (*p* < 0.01). Analyses using Egger’s test based on participants 45 years of age or older and less than 45 years old (p values were 0.4142 and 0.3639, respectively) indicated the absence of publication bias.

#### Subgroup analysis based on year of study implementation

**Fig. 10:**
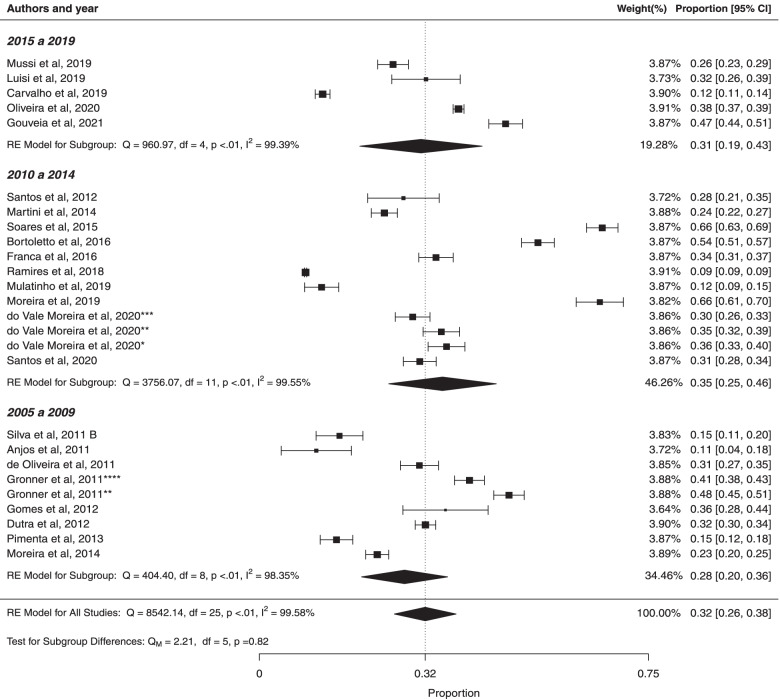
Forest plot of prevalence of metabolic syndrome according year of study implementation in Brazilian population

The prevalence of metabolic syndrome among the studies that was implementation in 2015–2019 was 31% (95% CI: 19%; 43%) with high heterogeneity (${I}^{2}$ = 99.39%; Cochran Q-statistic *p* < 0.01; Fig. 10). The studies that were impletmentation in 2010–2014 presented the prevalence of metabolic syndrome in 35% (95% CI: 25%; 46%) with high heterogeneity (${I}^{2}$ = 99.55%; Cochran Q-statistic *p* < 0.01). The studies that were impletmentation in 2005–2009, weighted pooled prevalence of metabolic syndrome was 28% (95% CI: 20%; 36%), with high heterogeneity (${I}^{2}$ = 98.35%; Cochran Q-statistic *p* < 0.01). There was not statistically significant difference between studies based on year of study implementation (*p* = 0.82). In addition, there was high heterogeneity in prevalence estimates across studies (*p* < 0.01). Analyses using Egger’s test based on years of study implementation in 2015—2019, 2010 – 2014 and 2005 – 2009 (*p* values were 0.7205, 0.3082 and 0.5632, respectively) indicated the absence of publication bias.

#### Meta-regression analyses

To assess the sources of heterogeneity, we performed a meta-regression. In these analyses, year of implementation and age of participants variables were not significantly associated with heterogeneity, *p* = 0.5291, *p* = 0.7369, meta-regression coefficient 0.0051, 0.0025 and confidence interval 95% CI -0.0108; 0.0211, -0.0122; 0.0172 respectively (Table 3).

**Table 3:** Results of meta-regression for the prevalence of metabolic syndrome

Covariate	Meta-regression coefficient	95% confidence interval	*P* value
Year of implementation	0.0051	-0.0108—0.0211	0.5291
Age	0.0025	-0.0122—0.0172	0.7369

## Discussion

We have conducted this review including studies performed in the last decade to obtain a comprehensive estimate of burden of MS in Brazilian adult population. In total, we analysed data from 26 studies that involved 84,522 participants. We have also captured the gender distribution, habitat differences, geographical region, criteria used to define metabolic syndrome, age of study participants and year of the study implementation estimates to find any significant difference in the estimates of MS.

Our meta-analysis revealed that the pooled estimate of MS prevalence among subjects in Brazil was 33%. This estimate was higher than the prevalence of 29.6% observed in Brazil in 2013 [14] and approached the worldwide prevalence of 20–25% [3]. The prevalence was also higher than that found in Malaysia (27.5%) [50], in the Philippines (19.7%) [51], Bangladesh (30.0%) [19] and Nigeria, whose prevalence was 31.7%, 27.9% and 28.1%, according to the definitions of WHO, ATPIII and IDF, respectively [52]. In the South Asia region, the weighted average prevalence of metabolic syndrome was 14.0% according to the WHO definition, 26.1% according to ATPIII, 29.8% according to the IDF and 32.5% according to the criteria modified from NCEP-ATPIII [53]. On the other hand, the prevalence of metabolic syndrome found in this study was lower than that reported in countries like the USA, 34.5% (NCEP-ATPIII) and 39% (IDF) [54], Turkey, 36.6% (ATPIII) and 44.0% (IDF) [55] and Iran, 36.9% (ATPIII), 34.6% (IDF) and 41.5% (JIS) [56].

Despite the scarcity of studies on the prevalence of MS in Latin American countries, a meta-analysis encompassing these countries found an estimate lower than that found in our study, 24.5% [57]. In addition, countries such as Argentina and Venezuela also found values lower than those observed in this study, 27.5% and 26.1% respectively. [58, 59]. However, in Bolivia, a prevalence of 44.1% was observed [60] and in Peru, according to the definitions of the IDF and ATPIII, respectively, the prevalence of MS found was 35.3% and 28.2% [61]. The variation in the prevalence of MS around the world can be explained by marked cultural differences, which directly influence the lifestyle and consumption patterns of populations [62].

This study demonstrated increased waist circumference as the most frequent individual component of metabolic syndrome, and high blood pressure was shown the second most prominent metabolic syndrome component. The increased prevalence of abdominal obesity and high blood pressure on Brazilian population can have numerous causes. A study, with data from three cohorts, revealed that WC can predict the deterioration of other MS components, indicating that visceral obesity plays a central role in the development of the syndrome syndrome [63]. However, in countries such as Malaysia [50], Bangladesh [19] and Turkey [55], hypertension was reported as the most frequent component, representing 38%, 30% and 87.5% respectively, this large variation in the percentages of this component and in the waist circumference was also verified in our study. In Latin America [13], the prevalence of MS components varied greatly from one country to another. Overall, the component-weighted mean showed low HDL cholesterol as the most frequent component (62.9%), followed by hypertriacylglycerolemia (46.7%).

Environmental factors related to lifestyle, such as physical inactivity, unbalanced food and stress and are closely linked with higher prevalence of obesity and especially for the accumulation of adipose tissue in the abdominal region, tissue directly involved in the genesis of insulin resistance, which is a possible connection with MS. The decrease in insulin action in tissues, such as adipose tissue, leads to an increase in the inflammatory process, which induces this resistance. As a consequence, the accumulation of visceral adipose tissue in the body generating a high-risk cardiometabolic condition [64]. In addition, insulin promotes renal sodium reabsorption and, in hyperinsulinemic conditions, an exacerbation of this action is expected. In fact, comparing individuals with and without MS, it was observed that patients with the syndrome had significantly greater proximal sodium reabsorption, which can cause hypertension [65].

Study quality assessment shows that in many studies participants were not sampled in an appropriate way and the sample size was inadequate, which is a concern. Furthermore, some studies did not present sufficient coverage of the identified sample for data analysis. These criteria for evaluating the quality of studies demonstrate that some studies may have publication bias, which corroborates with evidente asymmetry on the funnel plot.

We observed considerable heterogeneity among the included studies to estimate the prevalence of MS in the Brazilian adult population. Prevalence of metabolic syndrome was the same in males and females, remaining with high heterogeneity. The wide variation in the prevalence of MS among populations in Brazil can be attributed to heterogeneity among the included studies. The country, in addition to being continental in size, has great epidemiology, demographic and socio-economic variability and multicultural characteristics, which makes the population very diverse, making it difficult to generalize the findings of this study in Brazil.

The subgroup analysis based on habitat, geographical region, criteria, age and year of study implementation was conducted in order to try to overcome this limitation. However, heterogeneity remained even after theses subgroup analysis. Hence, we tried to explain the between-study variability using meta-regression and found the potential sources of heterogeneity. However, meta-regression analyses did not indicate enough factors to explain the observed heterogeneity. We suggest that other factors such as lifestyle, alcohol and tobacco consumption, stress, diet and physical inactivity may influence MS heterogeneity. Furthermore, the small number of studies in some regions of Brazil did not allow for a more robust analysis of the prevalence in these areas.

Other studies that assessed the prevalence of MS in different countries also observed high heterogeneity among their data. Meta-analyses performed with data from the general population of Bangladesh [19], Iran [56], China [66], Middle East [67] and Mexico [18] showed heterogeneity greater than 90%. The study carried out in Bangladesh identified that the main source of heterogeneity was the geographical area of ​​the population. In the study conducted in China, the age of participants was associated with lack of homogeneity. In Mexico, the diagnostic criteria used were significantly associated with the heterogeneity. However, as in our work, the studies carried out in Iran and the Middle East, after performing analyzes by subgroups such as habitat, genus and diagnostic criteria, it was not possible to identify the source of this heterogeneity.

The high prevalence of MS found in this study has significant clinical and epidemiological implications, as, as mentioned, MS increases the risk of morbidity and mortality from cardiovascular diseases, in addition to being associated with a higher occurrence of diabetes [68], therefore, it directly interferes with the pattern and curve of illness in the country. This fact explains why the MS epidemic is considered a serious public health problem in Brazil, contributing to the exponential increase in spending in the health area. Thus, the results shown in this study are essential to guide strategies in the area of ​​primary care aimed at the prevention, screening and early treatment of MS.

Like other studies, this our systematic review and meta-analysis study has some limitations, like there is no uniformity of metabolic syndrome definitions, age groups, waist circumference and hyperglycemia cut-offs, and study settings in the studies included in the present review, resulting in limitations in comparability. Features also noted by de Carvalho Vidigal et al. in its systematic review carried out with Brazilian adults [14]. Furthermore, we could not estimate the role of important risk factors on MS such as physical activity and diet, since the studies included had not measured the effects of these factors. This review, we conduct some subgroup analyzes with limited data, such as MS prevalence based on age of participants, because many included studies did not present this information.

The major strength of the study is that we have tried to provide the first review with metanalisys on burden of MS among adult population in Brazil. In addition, the strength is the comprehensiveness of the process, which included a search of four different databases, well-defined inclusion/exclusion criteria, and extensive use of reference lists.

## Conclusion

This systematic review and meta-analysis evaluated the scientific literature on the prevalence of metabolic syndrome in Brazil. Our review indicates a high prevalence of MS in the healthy Brazilian adult population, when compared to numerous countries and with a world estimate. Furthermore, the high prevalence remained when we subdivided the data according to different criteria, such as diagnostic, gender, age and geographic area of subjects studied, which suggests urgent attention from both the clinical and public health viewpoint. Information on how MS and its components are distributed could provide a great deal of insight into MS and assist in the planning and implementation of future prevention and control programmes.

## Supplementary Information

**Additional file 1.**

## Data Availability

The datasets used and/or analysed during the current study available are available in the "supplementary file" section.

## Abbreviations

AHA: American Heart Association
BMI: Body mass index; DM2 type 2 diabetes mellitus
HDL: High density lipoprotein
IDF: International Diabetes Federation
IAS: International Atherosclerosis Society
IASO: International Association for the Study of Obesit
JBI: Joanna Briggs Institute
JIS: Joint Interim Statement
MS: Metabolic syndrome
NCEP-ATP III: National Cholesterol Education Program Adult Treatment Panel III
NHLBI: National Heart, Lung, and Blood Institute
PRISMA: Preferred Reporting Items for Systematic Review and Meta-analysis
WC: Waist circumference
WHF: World Heart Federation
WHO: World Health Organization
